# The effect of temperature on the viability of human mesenchymal stem cells

**DOI:** 10.1186/scrt350

**Published:** 2013-11-15

**Authors:** Yannis Reissis, Elena García-Gareta, Michelle Korda, Gordon W Blunn, Jia Hua

**Affiliations:** 1 Imperial College School of Medicine, Imperial College London, South Kensington, Exhibition Road, SW7 2AZ, UK; 2 RAFT Institute of Plastic Surgery, Mount Vernon Hospital, Northwood, Middlesex, HA6 2RN, UK; 3 UCL Institute of Orthopaedics and Musculoskeletal Science, Royal National Orthopaedic Hospital, Brockley Hill, Stanmore, HA7 4LP, UK

## Abstract

**Introduction:**

Impaction allograft with cement is a common technique used in revision hip surgeries for the last 20 years. However, its clinical results are inconsistent. Recent studies have shown that mesenchymal stem cells (MSCs) seeded onto allograft can enhance bone formation. This *in vitro* study investigates whether the increase in temperature related to the polymerisation of bone cement will affect the viability of human MSCs.

**Methods:**

The viability of human MSCs was measured after incubating them at temperatures of 38°C, 48°C and 58°C; durations 45 seconds, 80 seconds and 150 seconds. A control group was kept at 37°C and 5% carbon dioxide for the duration of the investigation (7 days). During the course of the study the human MSCs were analysed for cell metabolic activity using the alamarBlue™ assay, cell viability using both Trypan Blue dye exclusion and calcein staining under fluorescent microscopy, and necrosis and apoptosis using Annexin V and propidium iodide for flow cytometric analysis. A one-way analysis of variance with *a priori* Dunnett’s test was used to indicate the differences between the treatment groups, when analysed against the control. This identified conditions with a significant difference in cell metabolic activity (alamarBlue™) and cell viability (Trypan Blue).

**Results:**

Results showed that cell metabolism was not severely affected up to 48°C/150 seconds, while cells in the 58°C group died. Similar results were shown using Trypan Blue and calcein analysis for cell viability. No significant difference in apoptosis and necrosis of the cells was observed when human MSCs treated at 48°C/150 seconds were compared with the control group.

**Conclusions:**

The study suggests that human MSCs seeded onto allograft can be exposed to temperatures up to 48°C for 150 seconds. Exposure to this temperature for this time period is unlikely to occur during impaction allograft surgery when cement is used. Therefore, in many situations, the addition of human MSCs to cemented impaction grafting may be carried out without detrimental effects to the cells. Furthermore, previous studies have shown that this can enhance new bone formation and repair the defects in revision situations.

## Introduction

There is evidence to show that the incidence of revision hip surgery is still at a high level in the population, ranging from 16 to 51% [1-3]. Common indications are instability, dislocation, osteolysis and aseptic loosening of the implant. In addition, younger patients are now undergoing total hip arthroplasty and an ageing population means that more patients now require revisions to replace the failing implants.

In revision total hip replacement, impaction allograft with cement has been widely used for more than 20 years for filling bone defects and achieving initial implant stability, with good outcomes [4,5]. However, the regeneration of new bone within the defect site is often inconsistent and long-term clinical results are uncertain [6-9]. Ornstein and colleagues in 2006 carried out a 5-year follow-up of socket movement and loosening after revision with impacted morselised allograft bone and cement; of 17 first-time socket revisions, they reported that ‘five sockets showed signs of radiographic loosening at 5 years postoperatively’ [10].

Previous studies show that human mesenchymal stem cells (hMSCs), most commonly derived from bone marrow, can be proliferated and differentiated into bone lineage using tissue engineering techniques [11-13]. This has raised hopes of alternative stem cell therapies for the treatment of a number of degenerative conditions [14]. It was proposed that the addition of hMSCs onto allograft could restore the integrity of bone structure and enhance new bone formation. Recently, the roles of osteocyte and osteoblast signalling and of bone-derived scaffolding in encouraging the osteogenic differentiation of hMSCs have been reported [15,16]. Furthermore, in 2006 Korda and colleagues showed that these cells can survive the impaction forces imposed by the surgeon during impaction grafting [17]. After impacting the graft, the surgeon usually uses acrylic bone cement to fix the revision acetabular cup into the pelvis with the impacted bone. During polymerisation, acrylic bone cement generates heat in an exothermic reaction that is transmitted to the bone. hMSCs that are used in any tissue engineering application to enhance bone formation in impaction grafting would have to withstand the temperatures generated by the polymerisation reaction.

A number of studies have measured the peak temperatures at the bone–cement interface during cement polymerisation [18-20]. Gill and colleagues used thermal probes that were inserted into the femoral head to record the temperatures generated at the bone–cement interface during resurfacing arthroplasty; a median maximum temperature of 47.2°C was recorded [18]. In the literature, temperatures between 30 and 70°C have been recorded, and the duration of the peak temperatures varies from 30 seconds to 5 minutes. The discrepancy is mainly due to differences between *in vitro* studies, or those that use computer models, and *in vivo* studies; it seems that computer models often overestimate the rise in temperature of the bone to over 70°C in some cases [21]. This does not correlate with the temperatures encountered *in vivo*.

The purpose of this study is to investigate *in vitro* the viability of the hMSCs when they are exposed to different temperatures and durations. For this study, we simulated the peak temperatures previously recorded in *in vivo* studies when cement is used [18,20,22]. The hypothesis is that the hMSCs can survive temperatures and durations that occur during revision total hip replacement.

## Materials and methods

### Isolation of human mesenchymal stem cells

hMSCs were obtained from bone marrow cell aspirations harvested in the iliac crest of patients during a normal total hip replacement procedure (Royal National Orthopaedic Hospital, Stanmore, UK). Consent was given by all patients to obtain bone marrow during their surgery, and the consent forms from the donors were signed and witnessed. Ethical approval was gained from the NHS Health Research Authority, National Research Ethical Committee London – Stanmore (REC reference number 07/Q0506/10). Separate hMSC isolations were carried out for each experiment, from at least two different donors in total. Cells between passages 3 and 5 were used.

Cells at passage 1 were resuscitated by placing the cryovial stored in liquid nitrogen in a water bath at 37°C. Dulbecco’s modified Eagle’s medium (D6046; Sigma-Aldrich, Gillingham, UK) supplemented with 20% foetal calf serum (FCS4246; First Link, Wolverhampton, UK), penicillin (100 units/ml) and streptomycin (100 μg/ml; Gibco, Paisley, UK) (DMEM+) was also warmed in a water bath at 37°C. Then 1 ml DMEM + was gently added to the cells, which were left to stand for 5 minutes, before being transferred to a universal tube. Doubling volumes of DMEM + were then added to the universal tube, with 5-minute equilibration periods between each addition, until a total of 16 ml was reached. The cell suspension was centrifuged at 2,000 rpm for 5 minutes. The supernatant was discarded and the pellet of cells resuspended in 1 ml DMEM + using a gauge needle and a 1 ml syringe. The suspension was transferred to two T225 (225 cm^2^ growth area) polystyrene cell culture flasks (Corning, Tewksbury, Massachusetts, USA). Then 30 ml DMEM + were added to each flask and designated as passage 2. The cells were incubated at 37°C and 5% carbon dioxide (CO_2_) and regularly observed under a phase-contrast light microscope. The growth medium was changed every 3 to 5 days until the cultures reached 80 to 90% confluency.

### Characterisation of human mesenchymal stem cells

hMSCs were characterised by demonstrating their multipotent differentiation potential for two cell lineages: adipogenic and osteogenic [11].

#### Adipogenic differentiation

Cells at passage 3 were cultured under either adipogenic conditions or standard conditions for 21 days in 12-well plates (Orange Scientific, Braine l'Alleud, Belgium); 1 × 10^5^ cells/well were plated. Adipogenic medium was DMEM + with 1 μM dexamethasone (D2915; Sigma-Aldrich), 200 μM indomethacin (17378; Sigma-Aldrich), 500 μM 1-methyl-3-isobutylxanthine (15879; Sigma-Aldrich) and 10 μg/ml insulin (I0516; Sigma-Aldrich). Media were changed every 3 to 5 days. After 21 days all samples were stained with Oil Red O to check for the presence of lipids. hMSCs were washed with phosphate-buffered saline (PBS) and fixed in formal saline. They were covered with Oil Red O stain for 20 minutes, rinsed with distilled water and counterstained with Harris Haemalum for 3 minutes. Finally, they were rinsed with distilled water, air dried and observed under a phase-contrast light microscope.

#### Osteogenic differentiation

Cells at passage 3 were cultured under either osteogenic conditions or standard conditions for 28 days. Osteogenic medium was DMEM + with 0.1 μM dexamethasone (D2915; Sigma-Aldrich), 500 μM ascorbic acid (A4544; Sigma-Aldrich) and 10 μM β-glycerophosphate (G9891; Sigma-Aldrich); 5 × 10^4^ cells were seeded per well in both six-well and 12-well plates. Media in all wells were changed every 3 to 5 days. Cell proliferation was measured by DNA assay after 7, 14, 21 and 28 days of culture in either DMEM + or osteogenic medium. Cells were washed in PBS and lysed by adding autoclaved distilled water at 37°C. After being frozen at -70°C and thawed three times, samples were transferred to Eppendorf tubes and spun at 10,000 rpm for 10 minutes. Then 100 μl supernatant were loaded in triplicate for each sample into a FluoroNunc™ white 96-well plate. Samples of 100 μl of the standards, ranging from 20 to 0.3125 μg/ml DNA (Sigma Aldrich, Gillingham, UK), were also loaded in triplicate. Finally, 100 μl of 1.0 μg/ml Hoerchst 33258 dye (Sigma-Aldrich) were added to each sample and fluorescence was read at 460 nm using a plate reader (Fluoroskan Ascent, Labsystems, Ramsey, Minnesota, USA). The amount of DNA in the samples was calculated as micrograms. DNA was expressed as units per micrograms.

Changes in cellular morphology were regularly observed by phase-contrast light microscopy. Mineral matrix deposition by Von Kossa staining was checked at 21 days. hMSCs were fixed in methanol. They were covered with 1.5% silver nitrate (Sigma-Aldrich) and exposed to bright light for 1 hour. The cells were washed with distilled water before covering with 2.5% sodium thiosulphate (BDH, London, UK) for 5 minutes. Finally, they were counterstained in Neutral Red for 5 minutes, washed with distilled water, air dried and observed by light microscopy.

### Cell culture of human mesenchymal stem cells for heat treatment

hMSCs at passage 3 were seeded into 30 wells (six-well plates; Orange Scientific, Braine l'Alleud, Belgium) at a density of 30,000 cells/well, with 3 ml DMEM+. The plates were incubated at 37°C and 5% CO_2_ for 4 days.

### Heat exposure

Table 1 presents the temperatures and corresponding time periods that were tested in this study. The control plate was kept in the incubator at 37°C and 5% CO_2_ for the duration of the experiment (7 days).

**Table 1:** Conditions tested in the study, shown as temperatures and their corresponding time periods

	**Temperature**		
**Time (seconds)**	**38°C**	**48°C**	**58°C**
**45**	*n* = 3	*n* = 3	*n* = 3
**80**	*n* = 3	*n* = 3	*n* = 3
**150**	*n* = 3	*n* = 3	*n* = 3

**Figure 1:**
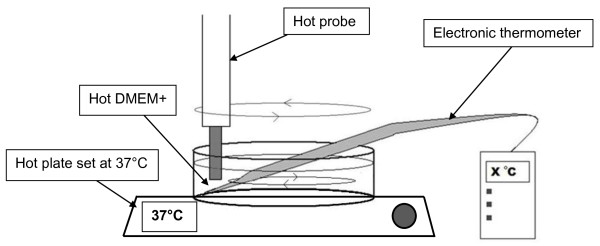
**Schematic illustration of the heating method used.** Heated medium was added to the wells in which human mesenchymal stem cells were cultured. The cells were kept on a hotplate at 37°C to replicate core body temperature. A hot probe was dipped into the medium to maintain the desired temperature. The medium was removed immediately once the desired time period had elapsed. DMEM+, Dulbecco’s modified Eagle’s medium supplemented with 20% fetal calf serum, penicillin and streptomycin.

For each experimental group, the same method of heating was used to control the temperature and duration (Figure 1). The six-well plate was kept on a hotplate to maintain the hMSCs at 37°C. Universal tubes with 13 ml DMEM + were heated to the required temperature in a water bath. When this temperature was reached the medium was immediately poured into the well containing the hMSCs; a timer, set at 45 seconds, 80 seconds or 150 seconds, was simultaneously started. An electronic thermometer was immersed in the medium and, if necessary, a hot probe was occasionally dipped into the medium and stirred to maintain the required temperature to an accuracy of ±1°C. The peak temperatures at 10-second intervals were monitored and recorded over the specified duration, after which the medium was immediately removed from the well. Then 3 ml DMEM + at 37°C was added to all the wells and the cells were put back into the incubator (37°C and 5% CO_2_).

### Analysis of cell metabolism by alamarBlue™

After heat exposure, 2 ml of a 10% dilution of alamarBlue™ solution (Alamar Biosciences, Sacramento, California, USA) in phenol-free medium (Dulbecco’s modified Eagle’s medium, D5921; Sigma-Aldrich) was added to the wells. Plates were incubated at 37°C and 5% CO_2_ for 3 hours. This assay was also carried out on the control plate kept at 37°C and 5% CO_2_.

After 3 hours, the plates were removed from the incubator. Then 100 μl alamarBlue™ solution was loaded in triplicate into a FluoroNunc™ white 96-well plate and absorbance was measured at 590 nm on a Flouroskan Ascent plate reader (Labsystems Inc., Ramsey, Minnesota, USA). All samples underwent continued culture for 7 days. After 0, 1, 2, 3 and 7 days, analysis of cell metabolic activity using alamarBlue™ assay was repeated. hMSCs were washed with 1 ml cold PBS between alamarBlue™ analyses and were maintained at 37°C and 5% CO_2_.

### Scanning electron microscopy study

Table 2 summarises the groups that underwent scanning electron microscopy (SEM) analysis (*n* = 2). A control sample, kept at 37°C and 5% CO_2_, was also analysed. Samples were fixed for SEM after 0 (immediately after heating), 3 and 7 days.

**Table 2:** Samples that underwent scanning electron microscopy analysis to assess changes to cell morphology

	**Temperature**		
**Time (seconds)**	**38°C**	**48°C**	**58°C**
**45**	*n* = 2		
**80**		*n* = 2	
**150**			*n* = 2

The hMSCs were washed with PBS and fixed in 2.5% glutaraldehyde with 0.1 M sodium cacodylate buffer at pH 7.3 (Agar Scientific, Stansted, UK). They were then washed with 0.1 M sodium cacodylate buffer at pH 7.3 (Agar Scientific) and post-fixed in 1% osmium tetroxide (Agar Scientific) in 0.1 M sodium cacodylate buffer for 1 hour. Samples were dehydrated through a graded series of industrial methylated spirit (20 to 60%) and ethanol (70 to 100%). Finally, samples were treated for 2 × 4 minutes with hexamethyldisalazane (Agar Scientific) and left to dry. The samples were then mounted on stubs and gold/palladium sputter coated before observation under a scanning electron microscope (JEOL JSM 5500 LV, JEOL, Tokyo, Japan).

### Analysis of cell number and viability using Trypan Blue

hMSCs were seeded into six-well plates. The same method of heat exposure was used as before (Figure 1). Table 3 presents the groups that were tested, with a control group kept at 37°C and 5% CO_2_.

**Table 3:** Samples that underwent analysis of cell viability using the Trypan Blue exclusion dye

	**Temperature**	
**Time (seconds)**	**48°C**	**58°C**
**45**	*n* = 3	*n* = 3
**80**	*n* = 3	*n* = 3
**150**	*n* = 3	*n* = 3

The cells were trypsinised immediately after heating (0 days) using 1 ml of 0.5% trypsin-5.3 mM ethylenediamine tetraacetic acid · 4Na solution (15400; Gibco). A 1:2 dilution of the cells in Trypan Blue (T8154; Sigma-Aldrich) was made and transferred onto a cover-slipped haemocytometer. Using phase-contrast light microscopy, viable cells were identified as rounded and bright, whereas blue cells were considered nonviable. A cell count and the calculation of percentage viability were recorded. This was repeated after 1, 3 and 7 days.

### Analysis of cell viability using calcein staining

Table 4 presents the groups that underwent Calcein staining for cell viability 0, 1, 2 and 7 days after heating (*n* = 2). hMSCs were seeded into six-well plates and onto Thermanox™ coverslips (Nalge Nunc International, Penfield, New York, USA). The cells were heated using the same method as in the previous experiments (Figure 1). After 0, 1, 2 and 7 days, the samples were stained with 200 μl calcein and incubated for 1 hour. Fluorescence microscopy was used to identify viable cells (green) and necrotic cells (red).

**Table 4:** Samples that underwent calcein staining and subsequent fluorescence microscopy to assess cell viability

	**Temperature**	
**Time (seconds)**	**48°C**	**58°C**
**45**	*n* = 2	*n* = 2
**80**	*n* = 2	*n* = 2
**150**	*n* = 2	*n* = 2

### Annexin V and propidium iodide labelling for apoptosis and necrosis

hMSCs were seeded into a six-well plate at a density of 50,000 cells/well. Three wells were exposed to 48°C for 150 seconds using the same heating method as before (Figure 1) while the other three wells were used as a control. We followed the TACS® Annexin V Kit protocol (Trevigen Inc., Gaithersburg, Maryland, USA) to make the Annexin V incubation reagent, which was added to the samples 1 day after heating. Cells were removed from the well surface using 1 ml of 0.5% trypsin-5.3 mM ethylenediamine tetraacetic acid · 4Na solution (15400; Gibco), centrifuged and resuspended in the incubation reagent using a 10 ml syringe and needle. The cells were then left in the dark for 15 minutes. After adding binding buffer, the samples were processed using fluorescence-activated cell sorting flow cytometry (Becton-Dickinson FACScan, BD Biosciences, Franklin Lakes, New Jersey). The software used to acquire the data was CellQuestPro™ (BD Biosciences, Franklin Lakes, New Jersey). A dot plot was obtained for each sample, with viable cells appearing in the lower left quadrant (no positive staining for Annexin V or propidium iodide), early apoptotic cells staining for Annexin V in the lower right quadrant, necrotic cells staining for propidium iodide in the upper left quadrant, and late apoptotic cells staining for both Annexin V and propidium iodide in the upper right quadrant.

### Statistical tests

Statistical analysis was performed using SPSS.20 software on the results for the alamarBlue™ (Sacramento, California, USA) and Trypan Blue (Sigma Aldrich, Gillingham, UK) experiments. A one-way analysis of variance with *a priori* Dunnett’s test was used to indicate the differences between the treatment groups, when analysed against the control. Significant results (*P*≤0.05) indicated a marked difference in cell metabolic activity (alamarBlue™) and cell viability (Trypan Blue).

## Results

### Effects of heat on cell metabolic activity

**Figure 2:**
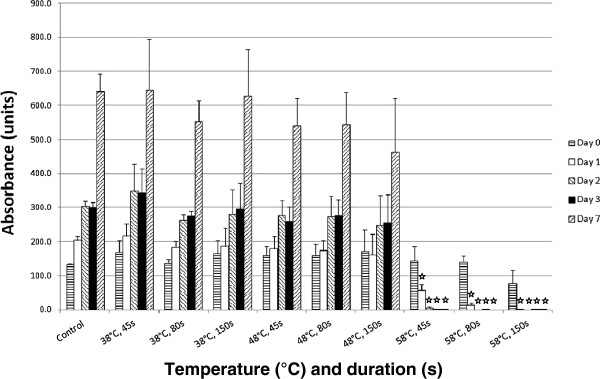
**Comparison of hMSC metabolic activity over 7 days.** The graph shows the absorbance of alamarBlue™ at 590nm for the different temperatures and durations tested. A higher absorbance corresponds to a higher level of metabolic activity, which is an indication of cell number. A lag phase of cell growth is indicated between days 2 and 3 for all samples up to 48°C/150 s, for which there were no significant changes in metabolic activity over 7 days when compared to a control. hMSCs heated to 58°C all eventually died. ^☆^*P*≤0.05, heated groups *vs*. control kept at 37°C/5% CO_2_.

Figure 2 shows the absorbance at 590 nm of alamarBlue™ over 7 days for the 10 groups. hMSC metabolic activity was tested 0, 1, 2, 3 and 7 days after heat treatment. A higher absorbance corresponded to a higher metabolic activity and is an indication of cell proliferation.

A similar pattern of cell metabolism and proliferation can be seen for the control and cells heated to temperatures up to 48°C. An increase in cell metabolic activity occurs over time as the cell number increases. However, the cells in the 58°C group showed very low proliferative activity. Cells heated to 58°C for all time periods showed a significant decrease (*P* ≤0.05) in metabolic activity from day 1, when compared with the control.

### Scanning electron microscopy analysis

**Figure 3:**
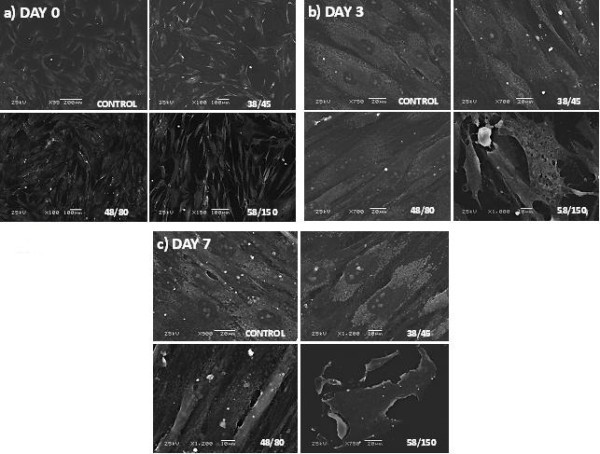
**Scanning electron microscopy of human mesenchymal stem cells.** A comparison of cell morphology of three different groups and a control at **(a)** 0 days, **(b)** 3 days and **(c)** 7 days after heating, indicating an increase in cell density over time for the control, 38°C and 48°C groups. Cells heated to 58°C rapidly died with signs of cell swelling and breakdown at day 3 **(b)**, leaving cell debris by day 7 **(c)**.

The SEM analyses indicate an increase in cell density over time for the control, 38°C and 48°C groups (Figure 3). The same morphology was observed for these three groups; cells displayed a flattened and elongated shape with multiple cytoplasmic processes of interaction between cells and with the material. A decrease in cell density over time was observed in the 58°C group, with only remnants of the cells seen after 7 days. The cells shrunk in size, and a disruption in cell integrity was noticed, with disrupted cell membranes and cytoplasmic processes of interaction.

### Analysis of cell viability using Trypan Blue

**Figure 4:**
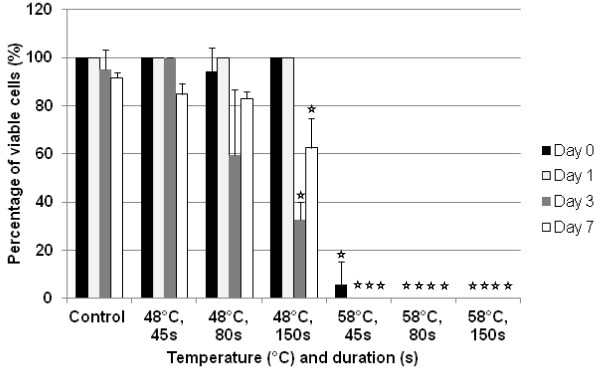
**Comparison of hMSC viability over 7 days.** Trypan Blue exclusion dye was used to calculate the percentage of viable cells in the 48°C and 58°C groups. The detrimental effect on cell survival after exposure to 58°C is shown. However, cell number seems to recover between 3 and 7 days after heat treatment at 48°C/80 s and 48°C/150 s. Error bars cannot be seen here for those tests in which no dead cells were seen in any population i.e. the standard deviation = 0. ^☆^*P*≤0.05, heated groups *vs*. control kept at 37°C/5% CO_2_.

Trypan Blue staining showed little change in the viability of the hMSCs in the control group and both 48°C/45 seconds and 48°C/80 seconds groups over 7 days (Figure 4). The control group showed a gradual decrease in percentage viability over time, typical for cells in culture. All of the samples heated to 58°C showed a significant detrimental effect on cell viability when compared with the control (*P*≤0.05).

**Figure 5:**
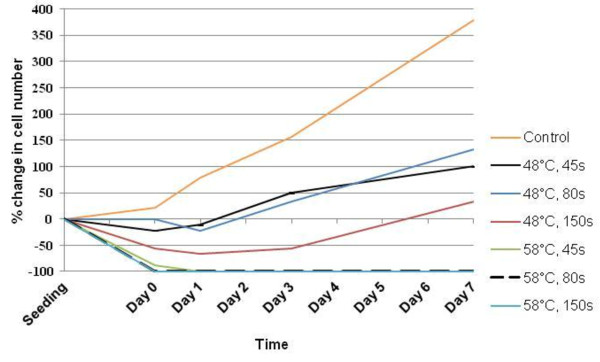
**Percentage change in cell number over time for all groups.** Trypan Blue exclusion dye was used to count the number of viable cells (white under the microscope) in the 48°C and 58°C groups. Recovery of human mesenchymal stem cell viability over 7 days is shown between the different groups; all groups excluding those heated to 58°C show net growth in cell number over the duration of the experiment.

The 48°C/80 seconds and 48°C/150 seconds groups showed a noticeable fall in percentage viability at 3 days, which was significant for the cells heated for 150 seconds (*P*≤0.05). This treatment group also showed statistical significance when compared with the control at 7 days. However, both samples revealed a subsequent increase in percentage viability between 3 and 7 days. This indicates a recovery of cell number in the culture, associated with the division of the remaining living cells. This is seen again in Figure 5, which shows the percentage change in the number of viable cells over 7 days; all samples excluding the 58°C group showed a net percentage increase in the number of viable cells (values for 58°C/80 seconds and 50°C/150 seconds were the same).

### Analysis of cell viability using calcein staining and light microscopy

**Figure 6:**
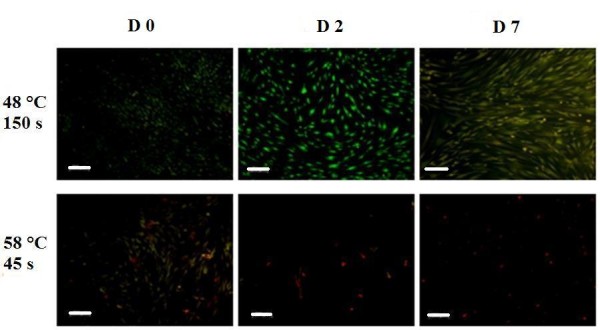
**Calcein staining for viable and dead cells over time using fluorescence microscopy.** Comparison between the 48°C/150 seconds and 58°C/45 seconds groups showing that the threshold for cell survival lies between these two conditions. Cells stained green are viable and cells stained red are dead. Scale bar = 200 μm.

A representation of the results obtained using Trypan Blue was achieved through calcein staining. Shown in Figure 6 are the 48°C/150 seconds and the 58°C/45 seconds groups, to highlight the limit for cell survival that lies in between these two conditions. hMSCs heated to 48°C/150 seconds display a healthy spindle morphology and normal cell growth over 7 days with viable cells staining fluorescent green. Heat treatment to 58°C for 45 seconds had an immediate deleterious effect on the hMSCs, seen after 0 days with the presence of dead cells, which stain red. After 7 days, only dead cells were seen. Heat exposure to 48°C for 45 seconds and 80 seconds showed similar results as for 150 seconds, with viable cells seen staining green. Exposure to 58°C for 80 seconds and 150 seconds had an immediate detrimental effect on cell viability, with red necrotic cells seen after 0 days.

**Figure 7:**
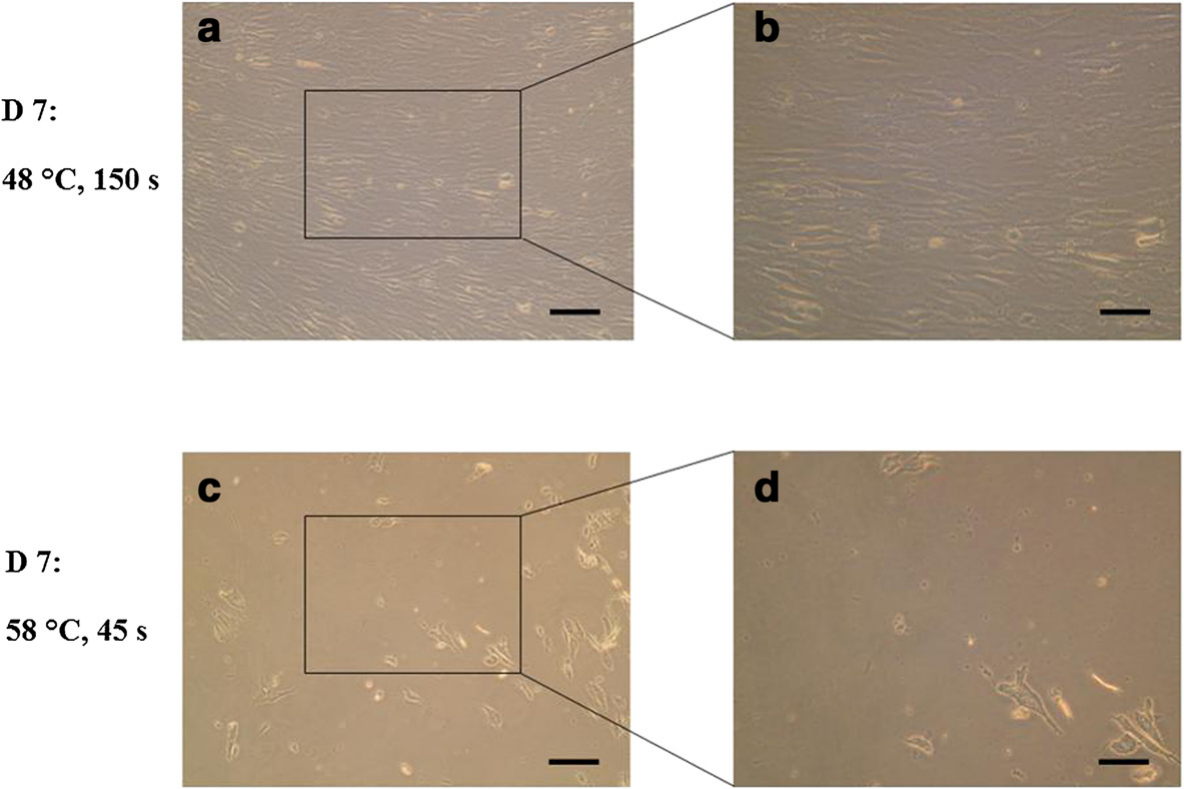
**Light microscope images of human mesenchymal stem cells.** Images taken at the end of the experiment showing the human mesenchymal stem cells in culture 7 days after heat treatment at 48°C for 150 seconds **(a), (b)** and at 58°C for 45 seconds **(c), (d)**. Normal spindle morphology with cell interactions can be seen after treatment at 48°C for 150 seconds, whereas cell damage is clear 7 days after heat treatment at 58°C for 45 seconds with only cell debris remaining. The threshold for cell survival lies between these two conditions of heat exposure. **(a)**, **(c)** Scale bar = 500 μm. **(b)**, **(d)** Scale bar = 250 μm.

Figure 7 shows images under the light microscope of hMSCs 7 days after being subject to the same conditions (48°C/150 seconds and 58°C/45 seconds), at magnification × 20 (Figure 7a,c) and magnification × 40 (Figure 7b,d). Normal spindle morphology can be seen after exposure to 48°C for 150 seconds (Figure 7a,b) whereas only cell debris is seen after exposure to 58°C for 45 seconds (Figure 7c,d).

### Annexin V and propidium iodide labelling for apoptosis and necrosis

**Figure 8:**
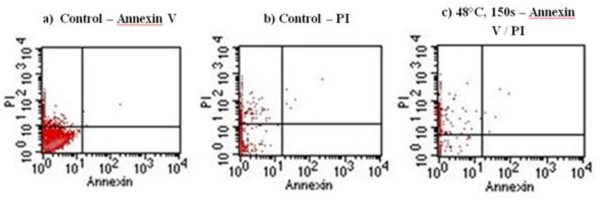
**Cell apoptosis and cell necrosis of human mesenchymal stem cells using Annexin V and propidium iodide staining.** Comparison between the unheated (control) and heated (48°C/150 seconds) groups using flow cytometry analysis: **(a)** control sample stained for Annexin V; **(b)** control sample stained with propidium iodide (PI) only; **(c)** heated sample stained for both Annexin V and PI. Annexin V staining is indicative of cell apoptosis; PI staining is indicative of cell necrosis.

A comparison to identify differences in cell apoptosis (using Annexin V staining) and cell necrosis (using PI staining) was carried out for a control group and for hMSCs heated to 48°C/150 seconds, which was the upper limit of the cells that survived heat treatment in the previous experiments. Flow cytometry was performed 1 day after heating. This early time point was used to pick up signs of apoptosis, which can be induced at an early stage, and to identify any necrotic cells that would otherwise have been lost on transferring the samples at a later stage. The graphs in Figure 8 show no evidence of staining for Annexin V alone in the lower right quadrant, suggesting there was no early apoptosis. In both the control and heated samples, very few cells had positive staining for both Annexin V and PI, seen in the upper right quadrant. This implies there were very few late apoptotic cells. However, a small population stained for PI in the upper left quadrant in both the control and the heated samples, a sign of necrosis. Little difference can be seen between the two groups, suggesting that the heat treatment to 48°C/150 seconds had no harmful effect on the hMSCs in terms of inducing apoptosis and necrosis, when compared with the control.

## Discussion

The use of impaction allograft with cement is a common technique used in revision total hip replacements for filling bone defects, and provides structural support to implants [6,10,23]. However, the regeneration of bone in the defect site is uncertain, with clinical results being inconsistent [5-9]. This may be due to the necrosis of the surrounding tissue caused by the heat generated at the bone–cement interface and resorption of the bone graft [18-20,24]. Previous studies have shown that addition of hMSCs onto allograft can enhance new bone formation [25-27].

In this study, hMSCs were heated up to a range of temperatures and durations that represent the highest temperatures generated at the bone–cement interface measured *in situ* in previous studies [18-22]. However, in clinical situations when impaction allografting is performed, a large number of hMSCs will not contact directly with the cement. Cells that are not in close proximity to the cement interface will experience lower temperatures; this was shown by Leeson and Lippitt in their study on the temperatures generated at different distances from the cement [22]. The experimental set up we used therefore represented the worst possible conditions.

The study showed that exposure to 58°C for as short as 45 seconds caused irreversible damage to cell integrity. This could be due to the fact that protein denaturation occurs at 56°C [20,26]. However, the viability and function of the cells were not severely affected by exposure to temperatures up to 48°C for 150 seconds; all six tests we used agreed that after 7 days these cells showed signs of growth and metabolic activity, as seen in the control sample, that were typical of hMSCs in culture. Interestingly, all of the samples up to this point, including the control group, showed evidence of the lag phase of cell growth typically between 2 and 3 days after heat treatment [17] shown by alamarBlue™ analysis of cell metabolic activity, which gives a proportional indication of cell number [28]. During these days some of the cells possibly died in culture, with the remaining cells each exhibiting a higher metabolic level, before exponential growth in cell number after 3 days; this hypothesis is reinforced by the Trypan Blue exclusion dye experiment, in which the 48°C/80 seconds and 48°C/150 seconds groups showed signs of a recovery in the cell number in culture after 3 days. The statistical significance in cell viability shown by Trypan Blue for the 48°C/150 seconds group at 7 days indicates that the cell function of some hMSCs is destroyed. Further tests, however, revealed that the remaining cells can survive and function normally up to day 7. Light microscope images of hMSCs in culture 7 days after heat treatment to 48°C for 150 seconds showed normal spindle cell morphology with healthy cell–cell interactions, with the cells confirmed to be viable by calcein staining at the same time point; the use of a fluorescent dye is often accepted to be more accurate in determining cell viability than Trypan Blue [29]. In this case it would have been desirable to continue monitoring cell growth over a longer period of time to see the full effect of heat treatment and to witness the extent of cell survival.

In addition, heat treatment to 48°C for 150 seconds was not shown to induce necrosis or apoptosis to any greater extent than in the control group. Annexin V staining for early-stage and late-stage apoptosis was negligible, the implication being that the hMSCs will not die due to apoptosis induced by heat exposure at a later stage postoperatively. Li and colleagues have shown that heat shock to 48°C for 10 minutes will cause irreversible damage to osteoblasts *in vitro* and induce late-stage apoptosis [30]. This duration of heat shock at 48°C exceeds that found at the bone–cement interface and so this damage is unlikely to occur when impaction allografting using cement is performed. Exposure of rabbit tibia to 53°C for 1 minute causes irreversible bone injury, after which healing occurs from the surrounding tissue [31]. This temperature may occur in some cases when cement is used, and may also affect the viability of hMSCs since this temperature lies within our critical temperature range of 48 to 58°C. However, heat shock protein 70 is shown to have a protective effect in osteoblasts when exposed to 45°C for 10 minutes, preventing apoptosis [30]. The protective effects of heat shock proteins in mesenchymal stem cells have not yet been studied in detail, and heat shock prior to exposure using cement may activate certain proteins that could make the cells more tolerant to higher temperatures.

Our previous study investigated the effect of the forces generated during impaction allograft in revision total hip replacement on the viability of hMSCs; the cells were found to withstand these forces [17]. The current study investigated the heat generated as a single factor that may affect the cells. In both studies the microplate alamarBlue™ assay was used to determine cell viability and proliferation, since alamarBlue™ has been shown to have a high specificity comparable with flow cytometry [32]. However, in clinical situations, there are multiple affecting factors that combine the impaction force, the rising temperature and the toxicity of cement monomer. Further study should therefore be carried out *in vivo* to test the combination of all these factors that can affect the viability of the hMSCs. In the clinical setting, heat exposure at 48°C for 150 seconds covers most of the temperature conditions when cemented impaction allograft surgeries are performed [18,19,24]. Our results therefore suggest that the heat generated during cement polymerisation will not affect the viability of the hMSCs, as the cells could withstand this temperature for this duration. Furthermore, the addition of hMSCs may enhance bone formation and repair the defects in revision situations. Therefore it is important to determine the ability of hMSCs to differentiate into new bone after heat exposure both *in vitro* and at a later stage *in vivo*[33].

## Conclusions

hMSCs seeded onto allograft can be exposed to temperatures up to 48°C for 150 seconds, whilst still holding onto their cell integrity and function. This result was shown through analysis post heat treatment for cell metabolic activity, cell viability, cell morphological changes and cell necrosis and apoptosis when compared with a control cultured under standard conditions. Exposure of the cells to this temperature for this time period is unlikely to occur during impaction allograft surgery when cement is used. The hMSCs were exposed only to temperatures found at the bone–cement interface in clinical situations, thus placing them in the worst possible conditions. Many cells would experience lower temperatures in real clinical situations. Therefore, in many cases the addition of hMSCs to cemented impaction grafting may be carried out without detrimental effects to the cells. Furthermore, previous studies have shown that this can enhance new bone formation and repair the defects in revision situations.

## Abbreviations

CO2: carbon dioxide; DMEM+: Dulbecco’s modified Eagle’s medium supplemented with 20% fetal calf serum, penicillin and streptomycin; hMSC: human mesenchymal stem cell; PBS: phosphate-buffered saline; SEM: scanning electron microscopy.

## Competing interests

The authors declare that they have no competing interests.

## Authors’ contributions

YR carried out the hMSC culture and heat treatment, participated in all experiments to analyse cell function and viability, and participated in the study design and wrote the manuscript. EG-G carried out SEM analysis and Annexin V/PI staining for flow cytometry, contributed to the study design and coordination, and carried out data analysis. MK aided in the study design and helped in the cell culture protocol, in particular the acquisition of the hMSCs. GWB participated in the study design and interpretation of data, and helped to draft the manuscript. JH participated in the study design and data analysis, and helped to draft the manuscript. All authors read and approved the final manuscript.
